# Identification of the Potential Key Circular RNAs in Elderly Patients With Postoperative Cognitive Dysfunction

**DOI:** 10.3389/fnagi.2020.00165

**Published:** 2020-06-23

**Authors:** Rui Gao, Chan Chen, Qi Zhao, Ming Li, Qiao Wang, Lu Zhou, Erya Chen, Hai Chen, Yue Zhang, Xingwei Cai, Changliang Liu, Xu Cheng, Shu Zhang, Xiaobo Mao, Yanhua Qiu, Lu Gan, Hai Yu, Jin Liu, Tao Zhu

**Affiliations:** ^1^Department of Anesthesiology and Translational Neuroscience Center, West China Hospital, Sichuan University, Chengdu, China; ^2^Department of Emergency Medicine, West China Hospital, Sichuan University, Chengdu, China; ^3^Department of Neurology, Institute of Cell Engineering, School of Medicine, Johns Hopkins University, Baltimore, MD, United States

**Keywords:** circular RNAs, microarray, postoperative cognitive dysfunction, aging, microRNAs

## Abstract

**Background:**

Postoperative cognitive dysfunction (POCD) is one of the severe complications after surgery, inducing low life quality and high mortality, especially in elderly patients. However, the underlying molecular mechanism of POCD remains largely unknown, and the ideal biomarker for clinical diagnosis and prognosis is lacking. Circular RNAs (circRNAs), as a unique class of non-coding RNAs, were characterized by its stability and conservativeness, serving as novel biomarkers in various diseases. Nevertheless, the role of circRNAs in the occurrence of POCD remains elusive.

**Methods:**

To investigate the differentially expressed circRNAs in the serum of POCD patients and its potential role in the development of POCD, we performed a circRNA microarray to screen the differentially expressed circRNAs in the serum samples from three patients of the POCD group and three paired patients of the non-POCD group. Subsequently, quantitative real-time polymerase chain reaction analysis (qRT-PCR) was utilized to verify the microarray data with the serum samples from 10 paired patients. Cytoscape software was used to construct the circRNA–miRNA–mRNA network for circRNAs with different expression levels as well as the target genes. Gene Ontology (GO) and Kyoto Encyclopedia of Genes and Genomes (KEGG) analyses showed the biological functions of the differentially expressed circRNAs target genes.

**Results:**

In total, we have analyzed 10,198 circRNAs through the microarray. Compared with the non-POCD patient group, there were 210 differentially expressed circRNAs with 133 upregulated and 77 downregulated in the POCD group (≥2-fold differential expression, *P* ≤ 0.05). The qRT-PCR confirmed 10 circRNAs with different expressed levels, and the results were consistent with the microarray findings. Among them, hsa_circRNA_001145, hsa_circRNA_101138, and hsa_circRNA_061570 had the highest magnitude of change. The GO analysis showed that the differentially expressed circRNAs were associated with the regulation of the developmental process, cell-to-cell adhesion, and nervous system development. The KEGG analysis showed that the target genes of circRNAs were enriched in the MAPK signaling pathway and RAS signaling pathway. According to the targetscan7.1 and mirdbV5 databases, the circRNA–miRNA–mRNA network was constructed, and these results provided a vital landscape of circRNA expression profile in POCD.

**Conclusions:**

Our study provides an essential perspective for the differential expression of circRNAs in POCD patients. Further studies need to be performed to explore their potential therapeutic roles in the development of POCD.

## Introduction

Postoperative cognitive dysfunction (POCD) is a severe and well-known complication following cardiac surgery with poor outcomes, especially in elderly patients (Steinmetz et al., 2009). What is worse, the neurocognitive function impairment after surgery can be persistent and result to a declined quality of life for the patients (Ballard et al., 2012). As one type of mild cognitive impairment, POCD could potentially increase the risk of Alzheimer’s disease (AD) (Grundman et al., 2004; Ward et al., 2012; Kapila et al., 2014). Therefore, many studies have been performed to explore its possible molecular mechanism. Recently, neuroinflammation, neuronal apoptosis, amyloid-Aβ (Aβ) deposition, and hyperphosphorylation of tau proteins have been reported as the main mediators in POCD (Xie et al., 2013; Chen C. et al., 2019; Li et al., 2019). Nevertheless, the pathogenesis of POCD remains elusive.

As a unique class of non-coding RNAs (ncRNAs), circular RNAs (circRNAs) are characterized by a covalently closed continuous loop, playing an important role in inhibiting the function of microRNAs (miRNAs), regulating the activity of RNA polymerase, binding the promoter sequence of corresponding genes, and influencing the expression of corresponding genes (Memczak et al., 2013; Bahn et al., 2015). Besides that, it has the characteristics of universality, conservativeness, and tissue specificity. Compared with linear RNAs, the stability of circRNAs is much higher so that it can exist stably in body fluid, serving as a valuable novel biomarker (Li P. et al., 2015; Li Y. et al., 2015; Xuan et al., 2016). Previous studies showed that circRNAs were involved in regulating various pathological processes of the central nerve cells and mediating the occurrence and the development of neurodegenerative diseases such as Parkinson’s disease and AD (Kumar et al., 2017; Piwecka et al., 2017). Meanwhile, the circRNA ciRS-7 has been found to promote the degradation of APP and BACE1 proteins and reduce the aberrant accumulation of β-amyloid peptide acting as a sponge adsorbent of microRNA-7 in AD (Shi et al., 2017). Furthermore, accumulative studies have reported similar pathomechanisms of persistent POCD as that of AD, such as the cell apoptosis, inflammation, and accumulation of misfolded proteins in the aging brain (Bilotta et al., 2010). However, the role of circRNAs in POCD is largely unknown.

Therefore, in our study, we performed circRNAs microarray to investigate the differential expression profiles of circRNAs from serum samples between the POCD and the paired non-POCD elderly patients undergoing cardiac valve replacement surgery under cardiopulmonary bypass. Subsequently, the miRNA binding sites and the related mRNAs were predicted by bioinformatics analysis. Besides that, we constructed the predicted circRNA–miRNA–mRNA co-expression network and investigated the principal functions and the pathways of target genes. These results may help to provide insights for clinical circRNA biomarkers and therapeutic targets for POCD.

## Materials and Methods

### Patients

The study was approved by the Ethics Committee of West China Hospital, Sichuan University, China (ChiCTR-OOC-16008289). Written informed consent was obtained from all the participants before the surgery. The patients scheduled to undergo an elective cardiac surgery under general anesthesia and cardiopulmonary bypass were enrolled in this study. The inclusion criteria were (1) more than 50 years of age, (2) American Society of Anesthesiologists physical status I–III, and (3) scheduled for cardiac surgery under general anesthesia. The exclusion criteria were (1) patients with second surgery, (2) ejection fraction less than 40%, (3) preoperative Mini-Mental State Examination (MMSE) scores less than 24, (4) patients with psychiatric disease or central nervous system disease, and (5) patients who were unable or rejected to complete preoperative neuropsychological testing.

### Neuropsychological Tests and Neurocognitive Evaluation

To assess patient cognitive function, a series of neuropsychological tests was performed on the day before surgery and 7 days after surgery by an experienced investigator. Various cognitive domains were evaluated by several neuropsychological tests including (1) MMSE, (2) Word Memory Test, a test used to assess the short-term and the long-term memory, (3) Digit Span Test, a test of the Wechsler memory scale used to measure concentration and attention, (4) Brief Visuospatial Memory Test—Revised (BVMT—R), a test to measure the immediate visuospatial and learning memory, (5) Symbol–Digit Modalities Test, a measure for psychomotor speed such that high scores indicated the better function, (6 and 7) BVMT—R Delayed Recall Test and BVMT—R Discrimination Index, the test used to estimate delayed recall abilities, (8) Trail Making Test, used to measure hand–eye coordination, concentration, and attention, and (9) Verbal Fluency Test, used to assess fluency and executive function. MMSE was performed to exclude patients with severe cognitive impairment. Then, all the subjects’ postoperative defects were defined as negative change scores, and the absolute value of each change score was greater than the standard deviation (SD) of the same cognitive test baseline score. A patient with two or more neuropsychological test defects was considered to be in the POCD group (Zhang et al., 2012; Saleh et al., 2015); otherwise, the patient was assigned to the non-POCD (NPOCD) group.

### Anesthesia and Surgery

All the patients underwent the same preoperative preparation, including corrective electrolysis disorder, maintenance of cardiac function, preoperative intramuscular injection of 3 mg scopolamine and 5 mg morphine, radial artery puncture catheterization, venous access, electrocardiogram, and oxygen saturation monitoring. We used midazolam (0.2–0.3 mg/kg), sufentanil (0.5–0.8 μg/kg), and cis-atracurium (2–3 mg/kg) to induction. The anesthesia was maintained by a continuous intravenous infusion of propofol and remifentanil, with an intermittent addition of midazolam, sufentanil, and cis-atracurium. Anesthesia machine was used for mechanical ventilation, such that the respiratory rate was 12 times per minute, and the tidal volume was 6–8 mlkg. After the anesthesia, central venous catheterization and manometry were performed. Mean artery pressure was controlled at 50–80 mmHg during a cardiopulmonary bypass. The perfusion dosage was 2.0–2.6 L/min/m^2^, and the hematocrit was 25–30%, with moderate hemodilution. After a cardiopulmonary bypass, protamine was used to neutralize heparin, such that the ratio was 1:1. The patients were transferred to the intensive care unit for further treatment after surgery.

### Blood Collection and Total RNA Isolation

All the patients signed an informed consent before the neuropsychological tests, and the blood samples were collected on the day before surgery and 7 days after surgery. Meanwhile, all the blood samples were examined, and it was made sure that they were not in the hemolysis state. After having been collected by one professional registered nurse, the whole blood was left undisturbed at room temperature for about 30 min. Following centrifugation at 3,000 × *g* for 10 min at 4°C, the supernatant (serum) was collected. We also confirmed that the color of the serum was clear and yellowish. Then, the supernatants (serum) were transferred to a refrigerator at −80°C for further analysis. The total RNA was extracted using the miRNeasy Serum/Plasma Kit (50) (Qiagen, Germany). The concentrations of the RNA samples were determined spectrophotometrically at 260, 280, and 230 nm by using a NanoDrop ND-1000 instrument.

### RNA Labeling and Assay Hybridization

The case–control matching of POCD and NPOCD groups was performed based on age (±2) and sex. Three pairs of serum samples were randomly selected for the microarray detection. Sample preparation and microarray hybridization were carried out in accordance with the standard protocol of Arraystar (Arraystar Inc., United States). In short, total RNAs were digested by RNase R (Epicentre, Inc., United States) to remove linear RNA and enrich circular RNAs. A random priming method was used to amplify and transcribe the enriched circular RNAs into fluorescent cRNAs (Arraystar Super RNA Labeling Kit; Arraystar). Then, the labeled cRNAs were purified by RNeasy Mini Kit (Qiagen, Germany). Five microliters of 10 × blocking agent and 1 μl of 25× fragmentation buffer were added in the labeled cRNAs, and then the mixture was heated at 60°C for 30 min. Finally, the labeled cRNAs were diluted by 25 μl of 2 × hybridizing buffer. Then, 50 μl of hybridizing solution was injected into the spacer slide and assembled on the circRNA expression microarray slide. The slides were incubated in an Agilent incubator at 65°C for 17 h. Agilent Scanner G2505C was utilized to wash, fix, and scan the hybridized arrays (Agilent Technologies, Santa Clara, CA, United States).

### CircRNA Microarray Data Analysis

The quantile standardization of the original data and the subsequent data processing were carried out by using the R software package through the log2 ratio. After quantile normalization of the original data, low-intensity filtering was performed, and the circRNAs with “P” or “M” marks (“All Target Values”) were retained for further analysis in three of at least six samples. When comparing the contour differences between the two groups, the “fold change” (i.e., the ratio of the group averages) between the groups for each circRNA was calculated. The statistical significance of the difference was conveniently estimated by *t*-test. The circRNAs with fold changes greater or equal to two and *P*-value ≤0.05 were chosen as the significant differential expression.

### Quantitative Real-Time PCR Validation of CircRNAs

Total RNA was extracted from serum using the miRNeasy Serum/Plasma Kit (50) (Qiagen, Germany). Quantitative real-time PCR (qRT-PCR) was conducted with an Eppendorf RT-PCR system (Hauppauge, NY, United States). We used *Caenorhabditis elegans*-miRNA 39 mimic (Ce-miR-39) as miRNeasy Serum Spike-In control. The specific primer pairs used in the study are listed in Table 1.

**TABLE 1:** Sequences for the primers used for the patients.

**Name**	**Bidirectional primer sequence (5′–3′)**	**Annealing temperature (°C)**
hsa_circRNA_ 001145	F: AATGGCCCTGGTAGCTTAGG	59.15
	R: CAAATCCCGATGGCCCACTT	60.68
hsa_circRNA_ 101138	F: CCTCTACCAGACCTCGCTGA	60
	R: GTACAGGGTGATGAGTCGGG	60
hsa_circRNA_ 030050	F: CAGCTCTTCCGGACTGTTCA	55
	R: CGCTGACCTTCCACTTTTGC	55
hsa_circRNA_ 061570	F: GGCAATCCATCCTCGGTGTA	55
	R: TCGTGGATGTATCCTTGTCGC	52.38
hsa_circRNA_ 401117	F: AGATGTGATCCTCCGGTTGG	55
	R: GTGACTTAGCATCCATGCCCT	52.38
hsa_circRNA_ 005537	F: AAACCTAGGAGAAGACCAGGCA	50
	R: CCACGGTCCAAACCATTCGG	60
hsa_circRNA_ 092522	F: ACCGGACAGAGTTTGATCGAC	52.38
	R: GGCATTTGGAGACTCCGCTA	55
hsa_circRNA_ 012989	F: ATACTTGCCAAATTGAGGCGG	59.5
	R: GCAGCATCAAAACCAGGTC	59.5
hsa_circRNA_ 005458	F: CATTCACAGCCAGAGTCGCT	59.5
	R: GATGTGCTGTGAGGGAGC	59.5
hsa_circRNA_ 050545	F: CATTCACAGCCAGAGTCGCT	59.5
	R: TCCAGTTGATTTAGCCCATTC	59.5
Ce-miRNA 39	F: ACACTCCAGCTGGGTCACCGGGTGTAAATC	59.80
	R: TGGTGTCGTGGAGTCG	59.73

### Annotation for CircRNA–miRNA Interaction

The circRNA–miRNA interaction was predicted with Arraystar’s (Rockville, MD, United States) miRNA target prediction software, according to TargetScan (Enright et al., 2003) & miRanda (Pasquinelli, 2012). The target miRNAs were predicted by miRNA support vector regression (mirSVR) algorithm to determine their score and rank the efficiency. Thus, five miRNAs were identified for each differentially expressed circRNA according to the mirSVR score. The network of the top five highest miRNA connecting to one circRNA was constructed.

### Prediction of CircRNA–miRNA–Target Gene Associations

We used targetscan7.1^1^ and mirdbV5^2^ databases to predict miRNAs’ target genes. We generally accept the overlapping results of two databases. To construct the interaction network, the cumulative weight context++ score <−0.3 and target score >70 were set as the cutoff. Then, Cytoscape software was used to construct the circRNA–miRNA–mRNA network for the top 10 differentially expressed circRNAs that we had validated as well as their predicted target genes. In addition, we performed gene ontology (GO) analysis^3^ to explore the potential functional roles of the target genes, including molecular functions (MF), cellular components (CC), and biological processes (BP). The potential biological pathway analysis related to circRNAs target genes was performed using the KEGG database. The *p*-value (EASE score, Fisher *P*-value, or hypergeometric *P*-value) indicated the significant correlation between the pathway and the conditions. The lower the *P*-value, the more significant the pathway is. *P* ≤ 0.05 showed that the GO terms and the KEGG pathways of the differentially expressed genes were significantly enriched. The microarray analysis was performed by Kang Cheng Bio-tech (Shanghai, China).

### Statistical Analysis

The categorical variables were presented as frequencies and analyzed using the chi-square test. The data are expressed as mean ± SD. All statistical data analyses were performed using GraphPad Prism 6.0. An analysis of the qRT-PCR validation between the POCD and the NPOCD groups was performed using Student’s *t*-test. The case–control matching was performed using SPSS 22.0 software. A rate value of less than 0.05 (*P* < 0.05) was considered as statistically significant.

## Results

### General Characteristics and Cognitive Functions of Participants

**FIGURE 1:**
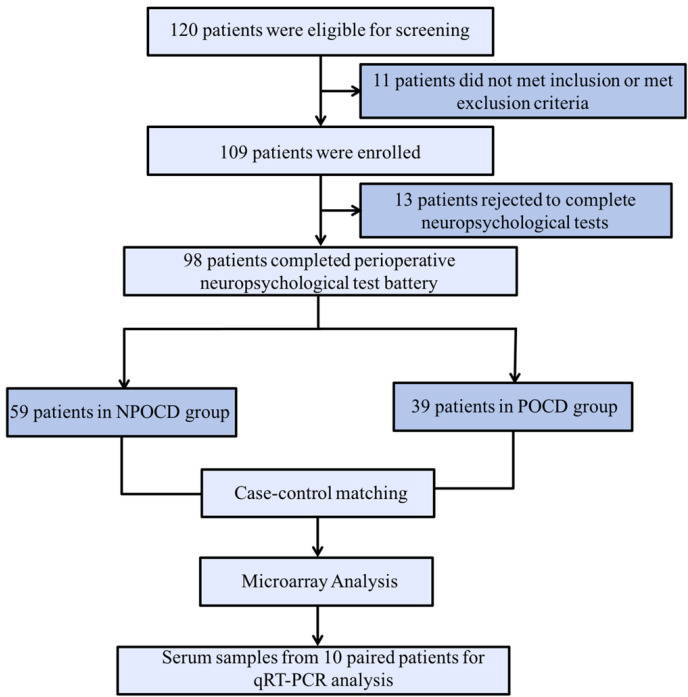
The process flow diagram of the experiment. POCD, postoperative cognitive dysfunction; NPOCD, the patients without postoperative cognitive decline; qRT-PCR, quantitative real-time PCR.

In total, there were 98 patients finally included who completed all perioperative cognitive tests and blood collections (Figure 1). Among them, 39 patients (39.8%) developed POCD, and 59 patients were assigned to the NPOCD group. No difference was observed between the two groups in terms of age, gender, body mass index, education level, and the baseline of each neuropsychological test (Table 2).

**TABLE 2:** General characteristics and cognitive functions of the participants from the postoperative cognitive dysfunction (POCD) and non-POCD groups at baseline.

**General characteristics and cognitive function scores**	**NPOCD (*n* = 59)**	**POCD (*n* = 39)**	***P* value**
Age (years)	58.7 ± 0.88	58.19 ± 0.96	0.7
Male	28 (47.4%)	17 (43.5%)	0.73
BMI (kg/m^2^)	23.00 ± 0.39	23.54 ± 0.54	0.4
Education	14 (23.7%)	11 (28.2%)	0.62
Cardiac function	2.78 ± 0.06	2.82 ± 0.08	0.69
Hypertension	11 (18.6%)	8 (20.5%)	0.82
Diabetes	2 (3.4%)	1 (2.6%)	0.82
Baseline Mini-Mental State Examination scores	26.91 ± 0.25	27.00 ± 0.32	0.89
Baseline Word Memory Test scores	10.53 ± 0.50	10.79 ± 0.49	0.98
Digit Span Test scores	15.76 ± 0.51	14.55 ± 0.58	0.61
Brief Visuospatial Memory Test—Revised (BVMT—R) scores	8.12 ± 0.55	8.37 ± 0.54	0.14
Symbol–Digit Modalities Test scores	26.74 ± 1.61	28.18 ± 1.68	0.55
BVMT—R Delayed Recall Test score	2.35 ± 0.26	3.02 ± 0.36	0.43
BVMT—R Discrimination Index score	10.38 ± 0.26	10.92 ± 0.49	0.29
Trail Making Test scores	68.09 ± 4.98	74.77 ± 8.42	0.48
Verbal Fluency Test scores	33.21 ± 1.24	37.32 ± 1.92	0.06

### Differential CircRNA Expression Profiles in POCD Patients’ Serum

**FIGURE 2:**
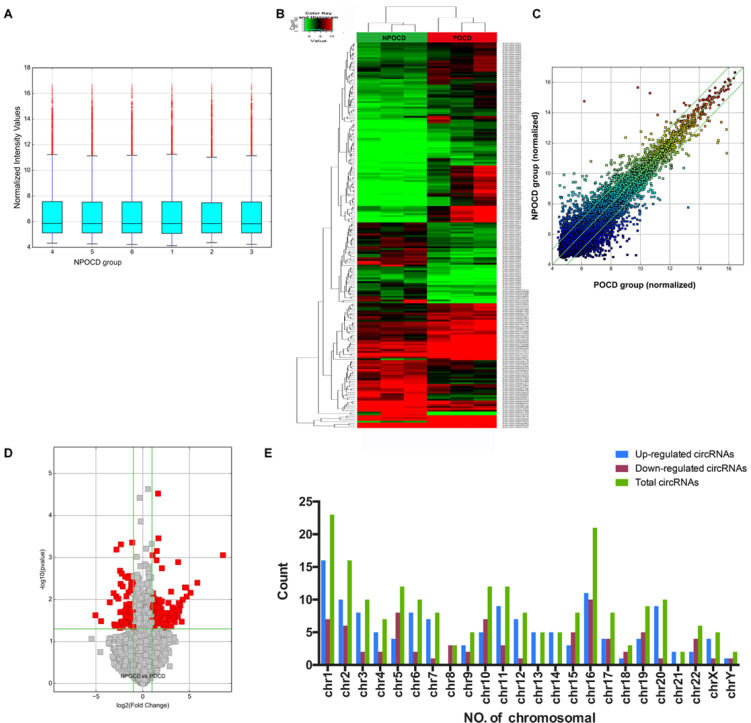
Differentially expressed profile of circRNAs and characterization between the serum of postoperative cognitive dysfunction (POCD) and non-POCD patients. **(A)** Box plots showing the distribution of circRNAs between the serum samples. **(B,C)** Hierarchical clustering plot showing the differentially expressed circRNA profiles in the six samples. “Red” represents the higher expression, while “green” represents the lower expression level. **(D)** Volcano plots visualizing the distinguishable circRNA expression. **(E)** Chromosomal distributions of circRNAs in the two groups.

Total RNAs were extracted from the serum samples of the two groups. The OD260/OD280 ratios of each sample were between 1.8 and 2.1, and the OD260/OD230 ratios were greater than 1.8, which demonstrated the RNA quality (Supplementary Figure 1). Using a human circRNA microarray, we drew the box plot to show the distribution of the intensities. We observed that the distribution of log2 ratios was similar after normalization in the tested samples (Figure 2A). The results of hierarchical clustering showed distinguishable circRNA profiling from six samples based on their expression level, indicating that circRNAs have different expression patterns in POCD patients compared with those in NPOCD patients (Figures 2B,C). The Volcano plot was performed to visualize the significant differences between POCD and NPOCD groups (fold change ≥2.0, *P*-value ≤0.05) (Figure 2D). Besides that, the distribution of the circRNAs differentially expressed in chromosomes showed that most of the circRNAs were transcribed from chr1, chr2, chr5, chr10, chr11, and chr16 and seldom from chr8, chr18, chr21, and chrY (Figure 2E). These data indicated that circRNAs have a different expression pattern in POCD serum compared with that in NPOCD serum. The microarray data also showed 210 circRNAs that were differentially expressed, among which 133 circRNAs were upregulated and 77 were downregulated in POCD patients’ serum. Among them, hsa_circ_001145 was most unregulated, and hsa_circ_005537 was most downregulated. Here we presented the top five upregulated and downregulated circRNAs (Table 3).

**TABLE 3:** Biological information for the top five upregulated and downregulated circRNAs.

**CircRNA ID**	**Fold change**	***P*-value**	**Chromosome**	**circRNA type**	**Best transcript**	**Gene symbol**
**Upgraduated**						
hsa_circRNA_001145	380.6	0.00088	chr20	Intronic	ENST00000317619	CPNE1
hsa_circRNA_101138	57.11	0.00399	chr12	Exonic	NM_130466	UBE3B
hsa_circRNA_030050	34.52	0.00692	chr13	Exonic	NM_172373	ELF1
hsa_circRNA_061570	25.71	0.00848	chr21	Exonic	NM_003024	ITSN1
hsa_circRNA_401117	24.89	0.00521	chr12	Exonic	NM_015394	ZNF10
**Downgraduated**						
hsa_circRNA_005537	33.66	0.02375	chr5	Exonic	NM_001790	CDC25C
hsa_circRNA_092522	22.01	0.03269	chr22	Exonic	uc003bhx.3	GRAMD4
hsa_circRNA_012989	11.52	0.01164	chr1	Exonic	NM_015017	USP33
hsa_circRNA_005458	8.67	0.00703	chr2	Exonic	ENST00000447760	AC114755.7
hsa_circRNA_050545	8.28	0.03876	chr19	Exonic	NM_005499	UBA2

### Amplification and Identity of Differentially Expressed CircRNAs

**FIGURE 3:**
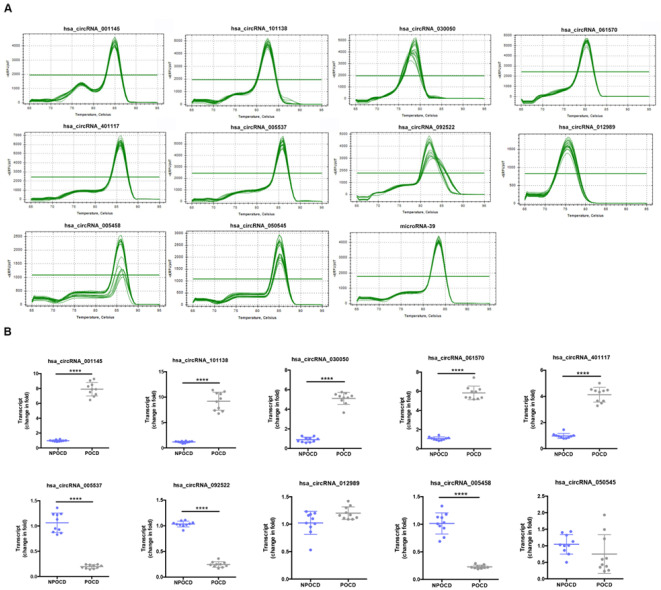
Amplification and identification of differentially expressed circRNAs. **(A)** The melt curves of the identified distinguishable expressed circRNAs. **(B)** qRT-PCR showing the expression levels of circRNAs between the two groups. Data are presented as means ± SD. ****P < 0.0001, *N* = 10.

Among all the patients, we randomly selected 10 pairs of serum samples from the POCD group and the NPOCD group. By using qRT-PCR, we verified 10 typically differentially expressed circRNAs. Meanwhile, the PCR results were identified by melt curve analysis such that the primers could specifically amplify the back-splice sites of circRNAs (Figure 3A). We found that the expression of hsa_circ_001145, hsa_circ_101138, hsa_circ_030050, hsa_circ_061570, and hsa_circ_050545 in the POCD group was significantly higher than those in the NPOCD group, while the expression of hsa_circ_005537, hsa_circ_092522, and hsa_circ_005458 was considerably downregulated in the POCD group. Therefore, the expression level of the abovementioned circRNAs was consistent with the analysis results from the microarray, validating the reliability of our microarray data (Figure 3B, ^****^P < 0.0001).

### Bioinformatics Analysis of the Predicted Network Genes for Differentially Expressed CircRNAs

**FIGURE 4:**
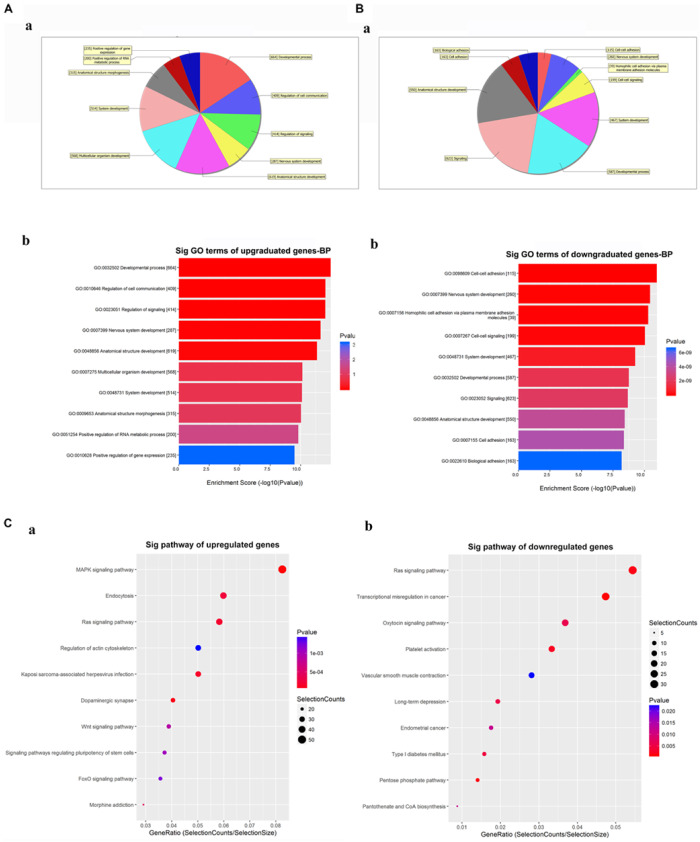
Gene Ontology (GO) and Kyoto Encyclopedia of Genes and Genomes (KEGG) analyses for the top five upregulated and downregulated circRNAs. **(A,B)** GO analysis showing the biological processes enriched by the target genes of the top five upregulated and downregulated circRNAs: **a**—classification of the predicted biological processes; **b**—the top 10 significantly enriched genes based on their enrichment scores. **(C)** KEGG pathway analysis showing the top 10 significantly enriched pathways and their scores. GO, gene ontology; Sig, significantly; BP, biological processes. Selection Counts, Count of the genes’ entities directly associated with the listed pathway ID; Selection Size, the total number of the genes’ entities.

The GO analysis for the differentially expressed circRNAs showed the top 10 significantly enriched target genes in terms of BP (Figures 4A,B), CC, and MF (Supplementary Figure 2). These results showed that the upregulated circRNAs had a strong relationship with the regulations of the developmental process (GO:0032502), cell communication (GO:0010646), nervous system development (GO:0007399), and so on (Figure 4A). Meanwhile, all downregulated expressed circRNAs were associated with cell-to-cell adhesion (GO:0098609), nervous system development (GO:0007399), and so on (Figure 4B). The KEGG analysis for differentially expressed circRNAs was performed to explore the top 10 significantly enriched pathways (Figure 4C). Especially, the target genes of upregulated circRNAs were mostly enriched in the MAPK signaling pathway, while those of downregulated circRNAs were enriched in the RAS signaling pathway (Supplementary Figures 3, 4). Of note is that these processes and pathways were reported to be associated with inflammation and apoptosis in POCD (Li et al., 2014; Wang et al., 2018).

### CircRNA–miRNA–mRNA Co-expression Network for the Differentially Expressed CircRNAs

**FIGURE 5:**
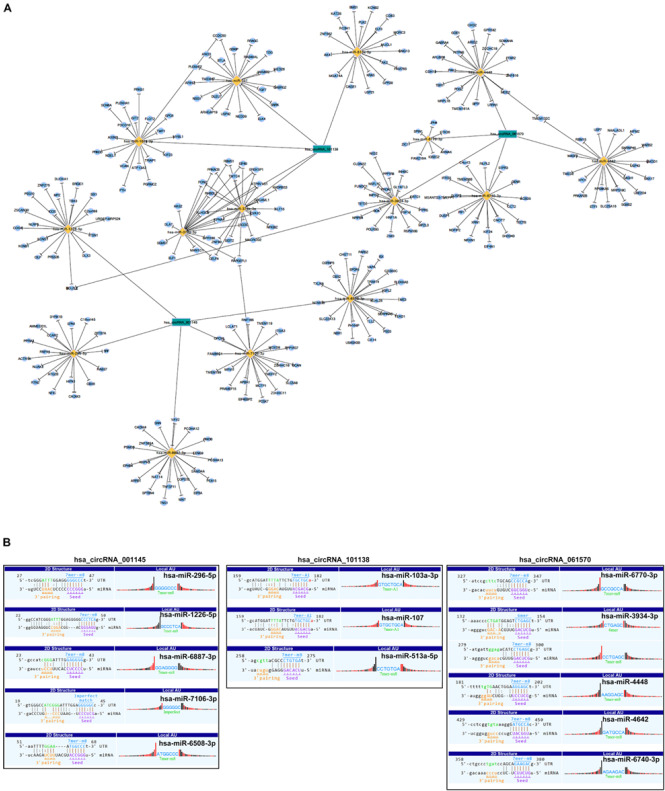
Representative circRNA–miRNA–mRNA network and sequence pairing predictions for circRNAs and miRNAs. **(A)** Based on the circRNA microarray profile results, the co-expression network drawn by Cytoscape software showing the dysregulated circRNAs, hsa_circ_001145, hsa_circ_101138, and hsa_circ_061570 (green nodes), having the highest magnitude of change and which were predicted to be functionally connected with their targeted miRNAs in the network. **(B)** Seed sequence matching predicted the direct interaction of the abovementioned circRNAs with their related miRNAs.

As there are specific binding sites of miRNAs in the circRNA sequence, circRNAs can interact with miRNAs by miRNA response elements. Five miRNAs with the highest mirSVR scores regarding the differentially expressed circRNAs, including the top five upregulated and downregulated circRNAs, are shown in Table 4. We assumed that the differentially expressed circRNAs act as a miRNA sponge to regulate its circRNA–miRNA–mRNA network. We then predicted the target genes of the top 5 miRNAs utilizing TargetScan & miRanda. Based on the overlapping results of targetscan7.1 and mirdbV5, there were 1,987 target genes for the top five upregulated circRNAs and 1,941 target genes for the top five downregulated circRNAs (Supplementary Figure 5). Based on our validation results of qRT-PCR, three circRNAs with the most significant change folds, hsa_circRNA_001145, hsa_circRNA_101138, and hsa_circRNA_061570, were used to construct the circRNA–miRNA–mRNA interaction network (Figure 5A). In contrast, an additional network diagram, considering all the rest of the validated circRNAs, was provided (Supplementary Figure 6). Utilizing the public databases (circBase^4^), the targeted miRNAs were screened, and the results were displayed according to their specific base pairing and seed sequence (Figure 5B).

**TABLE 4:** Predicted miRNA response elements of top five upregulated and downregulated circRNAs.

**CircRNA ID**	**Predicted miRNA response elements (MREs)**				
	**MRE1**	**MRE2**	**MRE3**	**MRE4**	**MRE5**
**Upgraduated**					
hsa_circRNA_001145	hsa-miR-296-5p	hsa-miR-1226-5p	hsa-miR-6887-3p	hsa-miR-7106-3p	hsa-miR-6508-3p
hsa_circRNA_101138	hsa-miR-376b-3p	hsa-miR-376a-3p	hsa-miR-513a-5p	hsa-miR-103a-3p	hsa-miR-107
hsa_circRNA_030050	hsa-miR-29b-1-5p	hsa-miR-7161-3p	hsa-miR-6868-3p	hsa-miR-142-3p	hsa-miR-19b-2-5p
hsa_circRNA_061570	hsa-miR-6770-3p	hsa-miR-3934-3p	hsa-miR-4448	hsa-miR-4642	hsa-miR-6740-3p
hsa_circRNA_401117	hsa-miR-4677-5p	hsa-miR-6733-3p	hsa-miR-199a-5p	hsa-miR-578	hsa-miR-657
**Downgraduated**					
hsa_circRNA_005537	hsa-miR-5009-5p	hsa-miR-6512-5p	hsa-miR-4753-3p	hsa-miR-6738-3p	hsa-miR-4760-3p
hsa_circRNA_092522	hsa-miR-3690	hsa-miR-615-5p	hsa-miR-422a	hsa-miR-1298-5p	hsa-miR-7641
hsa_circRNA_012989	hsa-miR-6792-3p	hsa-miR-8069	hsa-miR-4691-5p	hsa-miR-107	hsa-miR-103a-3p
hsa_circRNA_005458	hsa-miR-5708	hsa-miR-4701-3p	hsa-miR-765	hsa-miR-4522	hsa-miR-513a-5p
hsa_circRNA_050545	hsa-miR-6817-5p	hsa-miR-3170	hsa-miR-4769-3p	hsa-miR-4686	hsa-miR-513b-3p

## Discussion

In our study, we performed a circRNA microarray of the serum from clinical patients and found that the expression of circRNAs in POCD patients was significantly different from that in NPOCD patients. Among the 210 differentially expressed circRNAs, there were 133 circRNAs upregulated and 77 were downregulated. Next, we validated the typical circRNA expression levels in the two groups, and the results were consistent with the microarray analysis, showing the accuracy and the reliability of our microarray findings. Subsequently, our results showed the top five upregulated and downregulated circRNAs and predicted five miRNAs with the highest mirSVR scores for each circRNA, indicating the possible miRNAs involved in POCD. Then, we utilized GO and KEGG analyses to enrich the target genes of differentially expressed circRNAs in their biological functions and pathways. Finally, we constructed the circRNA–miRNA–mRNA interaction network for the three circRNAs with the most robust differential expression. Therefore, in our present study, through the human circRNA microarray and bioinformatics analysis, we have found the circRNAs with differential expression levels in POCD patients as well as the possible functions and pathways that their target genes might be involved in, thus trying to provide a preliminary perspective for the potential roles of circRNAs in POCD.

POCD is a serious perioperative complication with the main clinical manifestations of impaired memory, attention, language comprehension, and social skills after surgery in elderly patients, especially in heart, orthopedic, and abdominal surgery (Evered et al., 2011; Quan et al., 2019). It is of great clinical significance to find and screen out a molecule that can predict the occurrence of POCD at an early stage. Xie and his colleagues found that the ratio of Aβ to Tau protein in the cerebrospinal fluid (CSF) was closely related to neurocognitive dysfunction after surgery, which helped to identify POCD patients at an early stage (Xie et al., 2013). Besides that, the previous study also suggested that the CSF biomarkers in POCD were similar to that in AD. However, in clinical practice, the acquisition of CSF is invasive and difficult to implement. As a body fluid, blood can also carry a large number of valuable information molecules between the peripheral organs and the central brain. Therefore, searching for disease-related molecules based on blood detection and exploring the relevant regulation pathways are essential to find effective preventions for the development of POCD.

In recent years, accumulative studies have suggested that, in addition to the traditional roles of protein synthesis or enzyme-like catalytic activity, RNAs could also act as communicators between cells in physiopathological processes. Extracellular RNAs could be released from damaged cells under stress conditions, such as trauma, ischemia, and hypoxia (Kosaka et al., 2013). Additionally, the coding and the non-coding RNAs could transfer to the distant cells carried by extracellular microvesicles (Wei Z. et al., 2017). Furthermore, the protective role of ribonuclease (RNase), the counterpart of RNAs, was reported in several POCD models (Chen et al., 2015; Ma G. et al., 2017; Ma Y. et al., 2017). The application of RNase could reduce the amount of extracellular RNAs released from the damaged cells and then attenuate inflammation and apoptosis in the central nervous system. In our previous study, postoperatively increased double-stranded RNAs could activate toll-like receptor 3 and induce neuroinflammation in the hippocampus in an aged mouse model of POCD (Chen C. et al., 2019). Besides that, Chen and colleagues found that miRNA-146a could protect the cognitive decline induced by surgery trauma by suppressing neuroinflammation in mice (Chen L. et al., 2019). Also, emerging studies showed the differentially expressed lncRNAs and mRNAs and their potential roles in POCD disease as analyzed by microarray (Wei C. et al., 2017; Zhang et al., 2018; Li et al., 2019). Thus, it is necessary to investigate whether the circRNAs, a unique class of ncRNAs, participate in the pathogenesis of POCD.

CircRNAs exist ubiquitously in eukaryotic cells with high stability and evolutionary conservatism (Werfel et al., 2016). Additionally, it was reported that the expression level of circRNAs was much higher than that of corresponding linear mRNA in body fluid (Jeck et al., 2013). Because of their stability, conservativeness, and ubiquity, circRNAs were expected to become a new ideal marker for the diagnosis of diseases. Up to now, circRNAs were demonstrated to play vital roles in various conditions, including cancers, cardiovascular diseases, neurodegenerative diseases, and so on (Lukiw, 2013; Hancock, 2014; You et al., 2015; Wang et al., 2016; Zhou and Yu, 2017). However, the clinical value and the role of circRNAs in POCD have remained mostly unknown. Wang and colleagues performed a circRNA microarray based on the patients’ serum exosomes and predicted that circRNA-089763 might be the key circRNA in POCD development (Wang et al., 2019). Consistently, in our study, circRNA-089763 was also significantly upregulated, with 2.62 fold change in the microarray results, which was not shown in the manuscript. However, during the validation process, we verified a total of 10 circRNAs, which are all consistent with the microarray results. In comparison, they measured 15 circRNAs in which only the expression of circRNA-089763 was consistent with their microarray findings. Of note is that the pathomechanisms of diseases were complicated, while one gene would be regulated by a series of RNAs. The results of our microarray might help to expand the scope of other possible critical circRNAs involved in the pathogenesis of POCD and their potential communicable mechanism. Additionally, their study has focused on exosomal exRNAs, which might partially represent the differentially expressed circRNAs in the serum of POCD patients.

In general, circRNAs play the roles through negatively regulating the miRNAs upon target mRNAs as sponges or function with RNA binding protein (RBP) to regulate their parent genes (Chen, 2016). In this study, the highest mirSVRs of the top five upregulated and downregulated expressed circRNAs were identified by TargetScan and miRanda. Several miRNAs and target genes had been reported for their roles in neurodegenerative diseases. As shown in the circRNA–miRNA–mRNA network (Figure 5A), the predicted miRNA for hsa_circRNA_101138 included hsa-miR-107 in our study, and it was suggested to be the potential biomarker that would be downregulated early in AD (Swarbrick et al., 2019). Meanwhile, the numbers of potential target genes of hsa_circRNA_101138-hsa-miR-107 were also shown in the network, such as NEDD9 which was associated with AD genetically in Chinese (Xing et al., 2011) and GSKIP which functioned as anchoring proteins to strengthen the cAMP/PKA/Tau axis signaling during AD pathogenesis in CSF. Notably, Xie and his colleagues had found the import roles of Tau protein in CSF to identify POCD patients (Xie et al., 2013).

Besides that, hsa_circRNA_101138 could also regulate hsa-miR-376a-3p and hsa-miR-376b-3p targeting on the HAS2 gene shown in the network, its potential role of which was recently suggested in tau protein pathogenesis in AD related to cognitive dysfunction (Li et al., 2017). Additionally, hsa_circRNA_001145 was shown to potentially function with miR-1226-5p as sponge upon the ITSN1 gene in the circRNA–miRNA–mRNA network. At the same time, ITSN1 was also the parent target gene of hsa_circRNA_061570, as shown in Table 3, and might be regulated by hsa_circRNA_061570 through functioning with RBP. ITSN1 was reported to activate RAS-JNK signaling, thus damaging synaptic plasticity and reducing learning and memory functions in AD (Yarza et al., 2015). As previously reported, ITSN1 could predict neurodegenerative diseases early, such as AD and Down syndrome (Murphy et al., 1990).

Similarly, UBE3B was not only the parent target gene of hsa_circRNA_101138 but also the targeted gene of hsa-miR-1226-5p, which might be regulated by hsa_circRNA_001145. UBE3B was associated with speech ability and intelligence development (Cheon et al., 2019). Thus, one target gene involved in POCD might be regulated by several circRNAs through different mechanisms. From the network of circRNA–miRNA–mRNA, we also found that several target genes were co-targeted by different circRNAs. For example, BCL2L2 was the potential target gene of both hsa_circRNA_001145 and hsa_circRNA_061570, the differential expression of which was reported to induce systemic inflammation and cognitive decline (Pathak et al., 2019). Therefore, the circRNAs might function together through the communicable mechanism and the complicated networks, such as circRNA–miRNA–mRNA or RBP mechanism. In the present study, we just provide an expanded prospective for possible circRNAs and mechanisms promoting the development of POCD, and it is worthwhile to further investigate their potential roles.

Then, GO analysis showed that the target genes of these circRNAs were involved in nervous system development (GO:0007399) and synapse (GO:0045202) in terms of BP and CC, indicating their potential roles in influencing nervous development in the progress of POCD. A recent study has revealed that circRNAs could affect neuronal development and plasticity (van Rossum et al., 2016), which are also crucial in learning and memory (Amtul and Atta Ur, 2015). For example, EphB4, one potential target gene of hsa_circRNA_001145, was among several Eph gene families which were reported to be involved in the development of AD by influencing neuronal synapses formation (Dalva et al., 2000; Nolt et al., 2011; Zhang et al., 2016). Meanwhile, KEGG analysis enriched these genes in MAPK and RAS signaling pathways that were both reported to have vital roles in inflammation and apoptosis, inducing the cognitive impairment in POCD (Li et al., 2014; Wang et al., 2018). The parent gene of hsa_circRNA_061570, ITSN1, also was reported to activate RAS-JNK signaling, damaging synaptic plasticity and reducing learning and memory functions in AD (Yarza et al., 2015). The difference in our GO and KEGG analyses from Wang’s research was the gene quantities. More target genes regulated by several circRNAs were involved in POCD and would be enriched in different signaling pathways.

Our study also has some potential limitations. Firstly, we just provide an expanded prospective for possible circRNAs and potential mechanisms promoting the development of POCD. Thus, the blood samples were only collected at one time point after surgery when learning and memory dysfunction was observed. Further verifying of these screened-out circRNAs on several time points after surgery might provide more clinical significance for diagnosis or prediction. Secondly, the possible roles of circRNA in the development of POCD were based on bioinformatics prediction. Thus, further experiments *in vivo* would be performed to identify how these differentially expressed circRNAs initiate the POCD. Third, currently we could not distinguish the source of these differentially expressed circRNAs; more experiments might be performed to explore the relationship between POCD and the circRNAs originating from different organs or tissues.

In conclusion, our study has validated the significant differentially expressed circRNAs and predicted miRNAs and genes in the POCD patients. Our study could provide a preliminary perspective for circRNAs with different expressions and predict their potential participation mechanisms in POCD. In the future, further studies are still needed to investigate how these circRNAs function on genes regulating POCD development and become novel potential peripheral clinical biomarkers and therapeutic targets for POCD.

## Data Availability Statement

The raw datasets used and/or analyzed in the current study are have been uploaded in to Gene Expression Omnibus (GEO), and the GEO accession number is GSE147277.

## Ethics Statement

The studies involving human participants were reviewed and approved by Ethics committee of West China Hospital, Sichuan University, China (ChiCTR-OOC-16008289). The patients/participants provided their written informed consent to participate in this study. Written informed consent was obtained from the individual(s) for the publication of any potentially identifiable images or data included in this article.

## Author Contributions

RG, CC, TZ, and JL designed the experiments. RG, QZ, LZ, QW, YQ, YZ, XCa, XCh, CL, and HC performed the experiments. RG, CC, XM, SZ, TZ, and EC analyzed the data. RG, CC, TZ, LG, XM, and HY wrote the main manuscript text. All the authors reviewed the manuscript.

## Conflict of Interest

The authors declare that the research was conducted in the absence of any commercial or financial relationships that could be construed as a potential conflict of interest.

## Footnotes

1 http://www.targetscan.org/vert_71/

2 http://mirdb.org/miRDB/

3 http://www.geneongoloty.org/

4 http://www.circbase.org
