# Supplementary material for: Identification of the Potential Key Circular RNAs in Elderly Patients With Postoperative Cognitive Dysfunction
Source: Front Aging Neurosci. 2020 Jun 23;12:165. doi: 10.3389/fnagi.2020.00165 (PMC7324535; doi:10.3389/fnagi.2020.00165)
Supplement: Supplementary file 1 [file Table_1.DOCX]

**
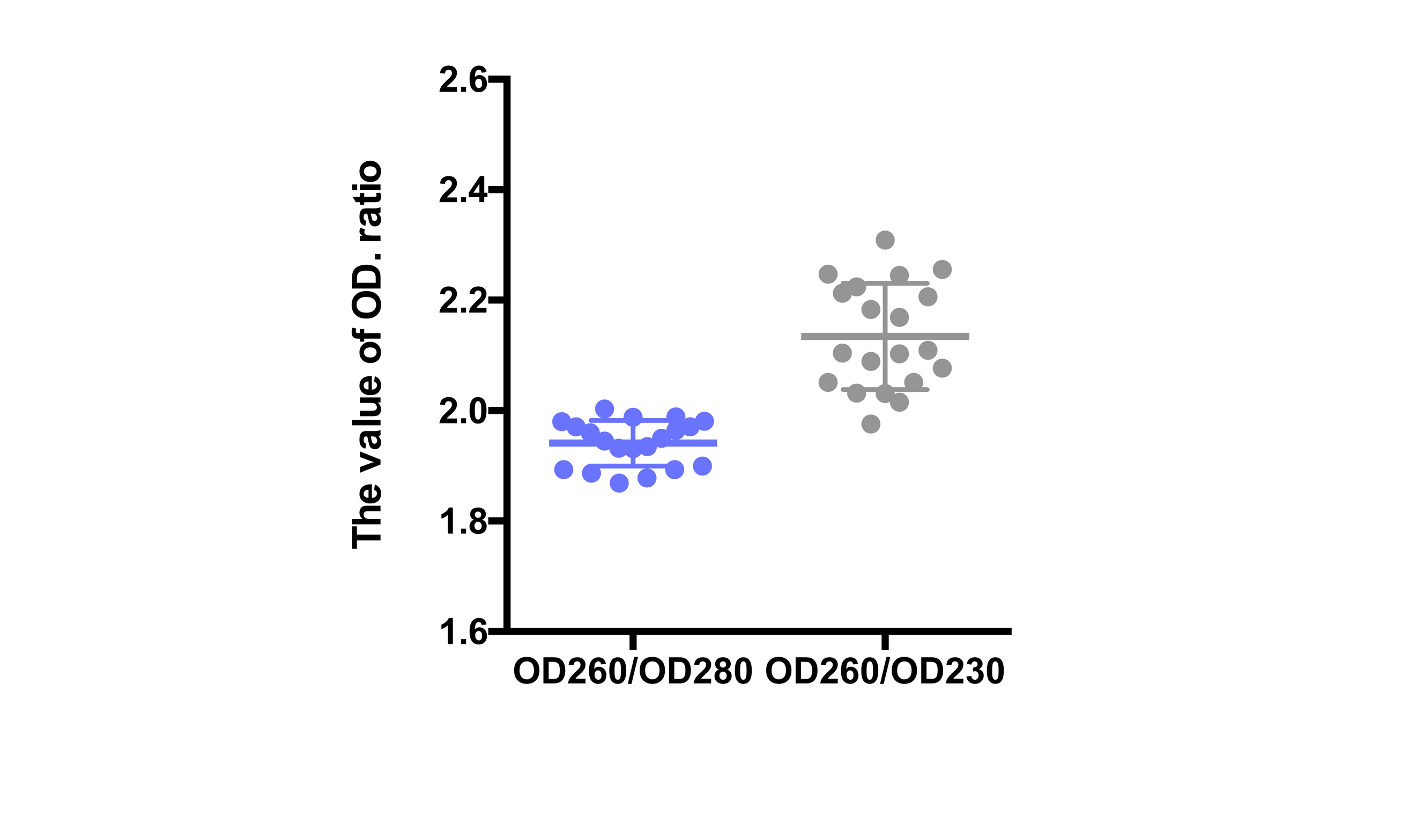
Supplementary materials**

**
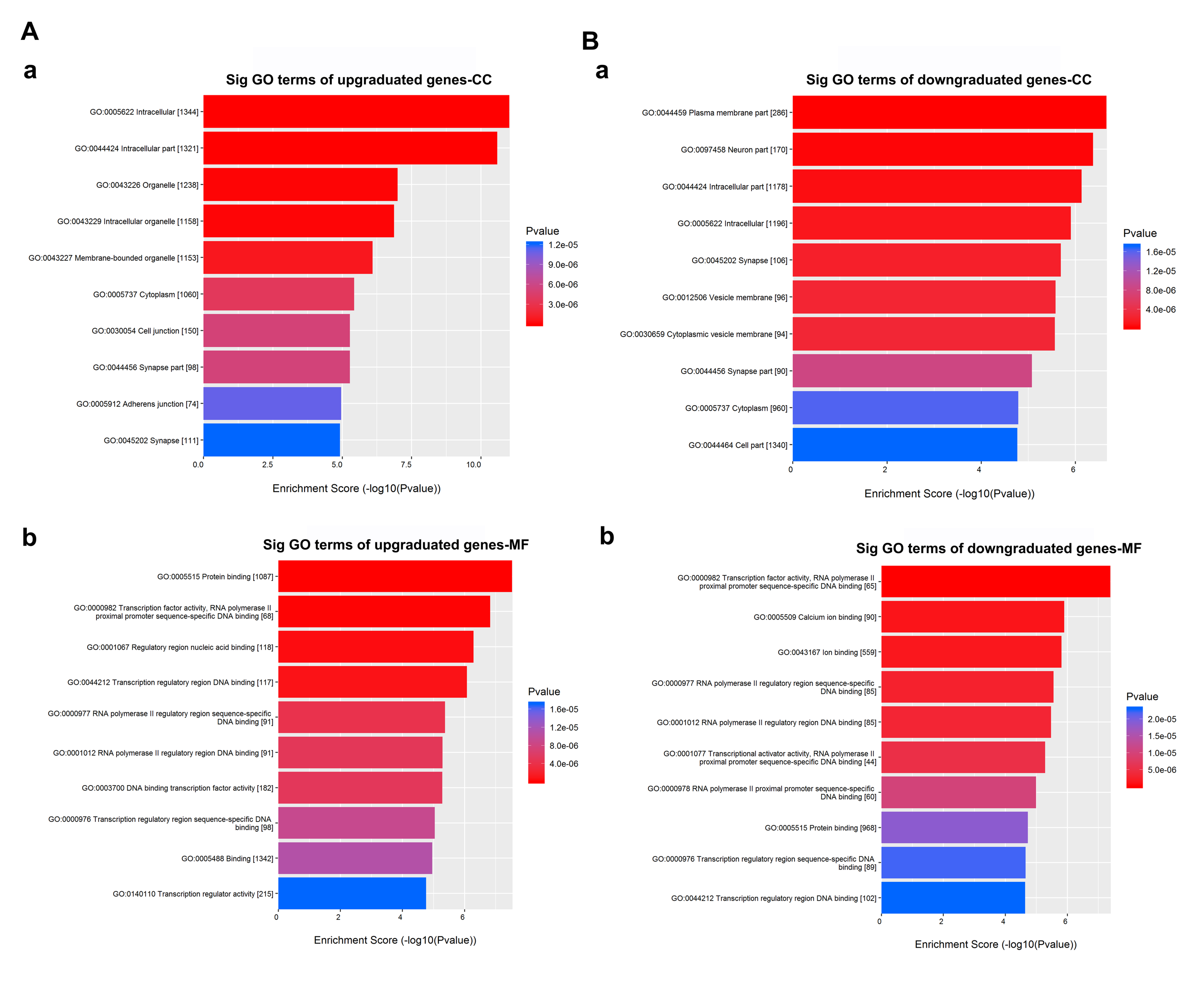
Figure 1.** Validation of serum samples’ RNA quality. **(A)** OD260/OD280 ratios should be between 1.8 and 2.1, OD260/OD230 ratios should be more than 1.8.

**Figure 2.** Gene ontology (GO) enrichment analysis for top 5 upregulated and downregulated circRNAs. **(A)** GO enrichment analysis for upregulated circRNAs target genes in terms of cellular components (CC) and molecular functions (MF). **(B)** GO enrichment analysis for upregulated circRNAs target genes in terms of cellular components (CC) and molecular functions (MF).

**
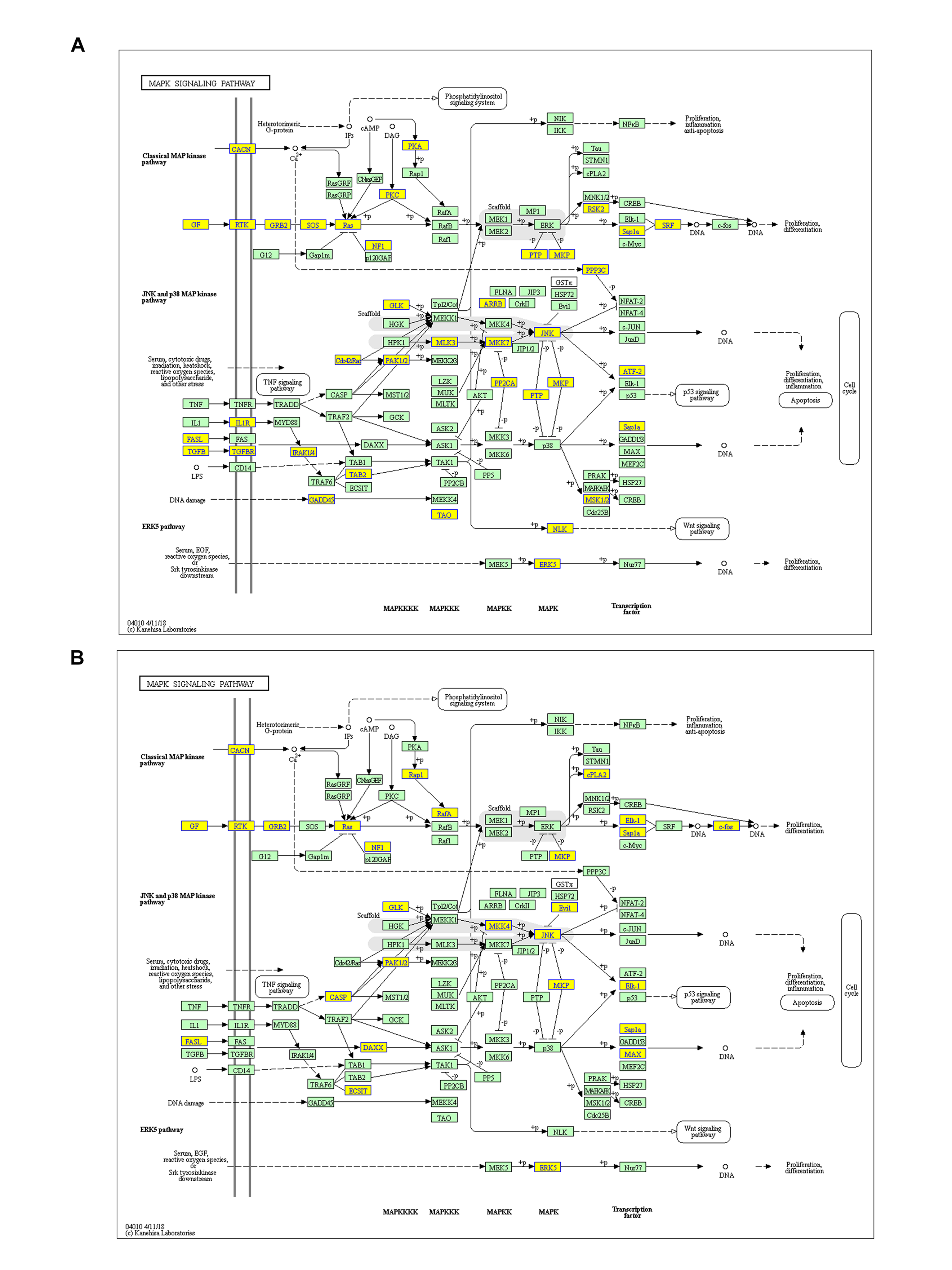
**

**Figure 3.** MAPK signaling pathway analysis for predicted circRNAs. **(A)** MAPK signaling pathway analysis for upregulated expressed circRNAs**. (B)** MAPK signaling pathway analysis for downregulated expressed circRNAs. The image was obtained from KEGG (http://www.kegg.jp/kegg/kegg1.html).

**
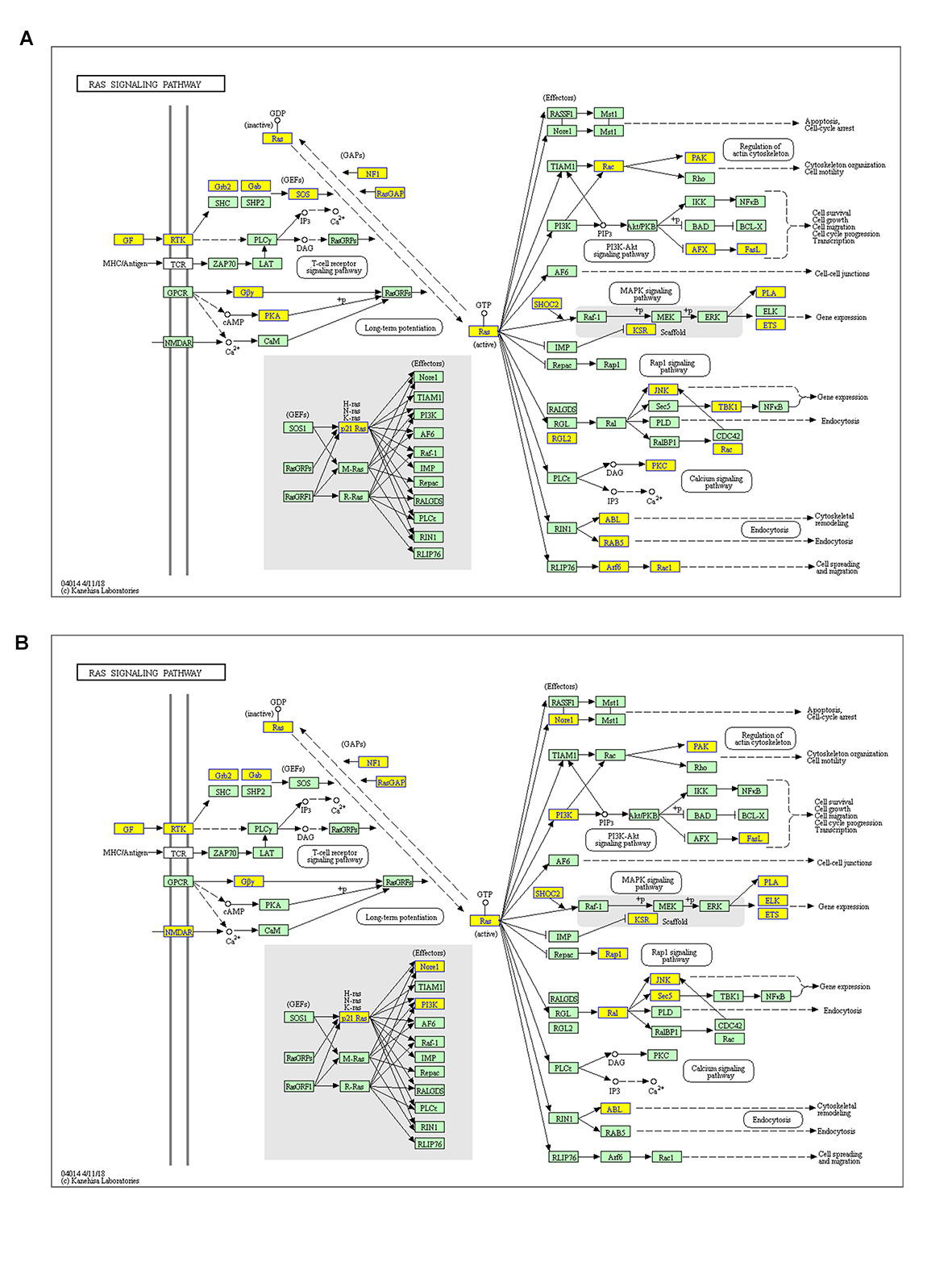
**

**Figure 4.** RAS signaling pathway analysis for predicted circRNAs. **(A)** RAS signaling pathway analysis for upregulated expressed circRNAs. **(B)** RAS signaling pathway analysis for downregulated expressed circRNAs. The image was obtained from KEGG (http://www.kegg.jp/kegg/kegg1.html).

**
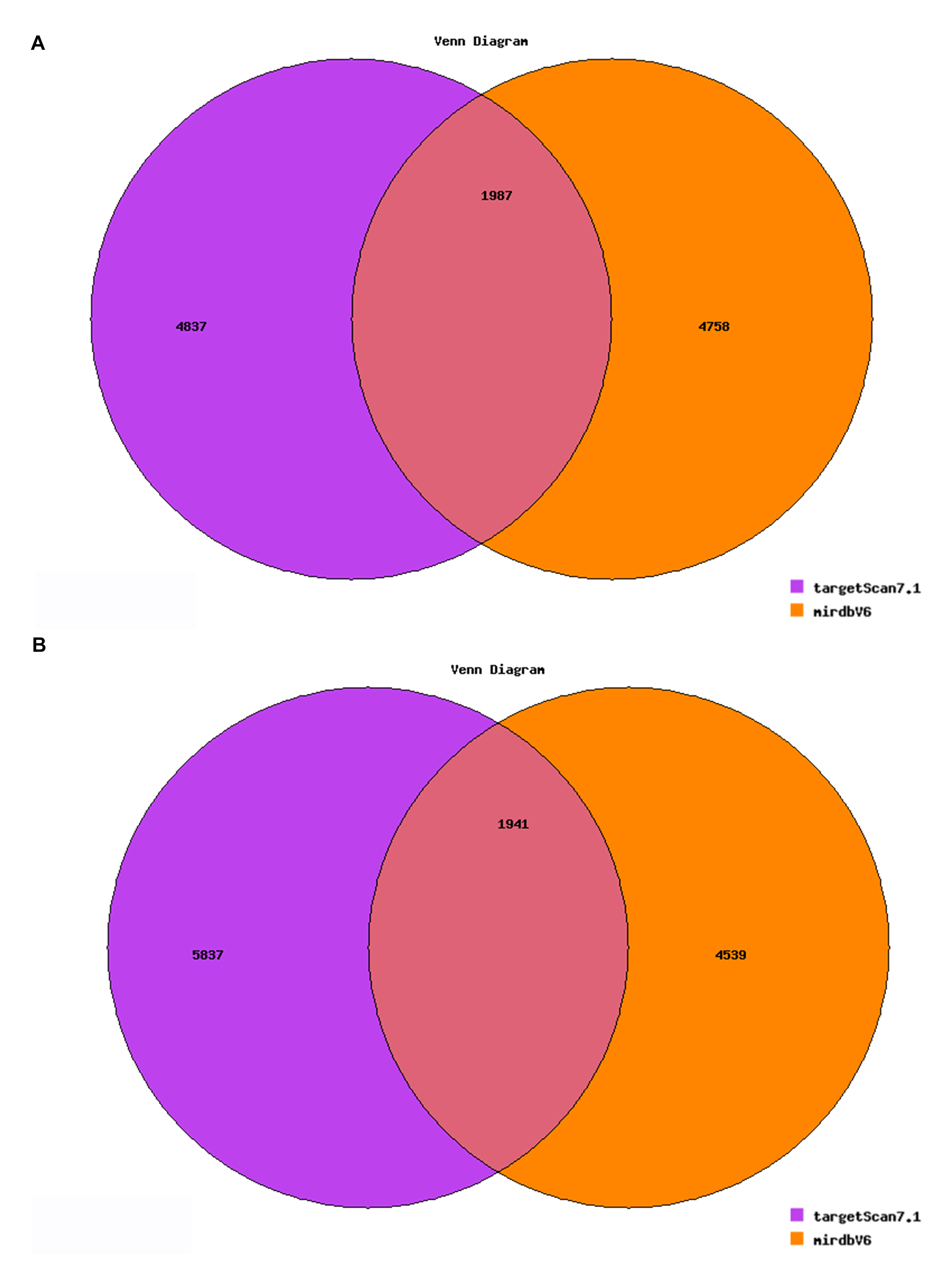
**

**Figure 5.** Venn plot for predicting the number of mRNA target genes based on targetscan7.1 and mirdbV5. **(A)** Top 5 upregulated circRNA target genes. **(B)** Top 5 downregulated circRNA target genes.

**
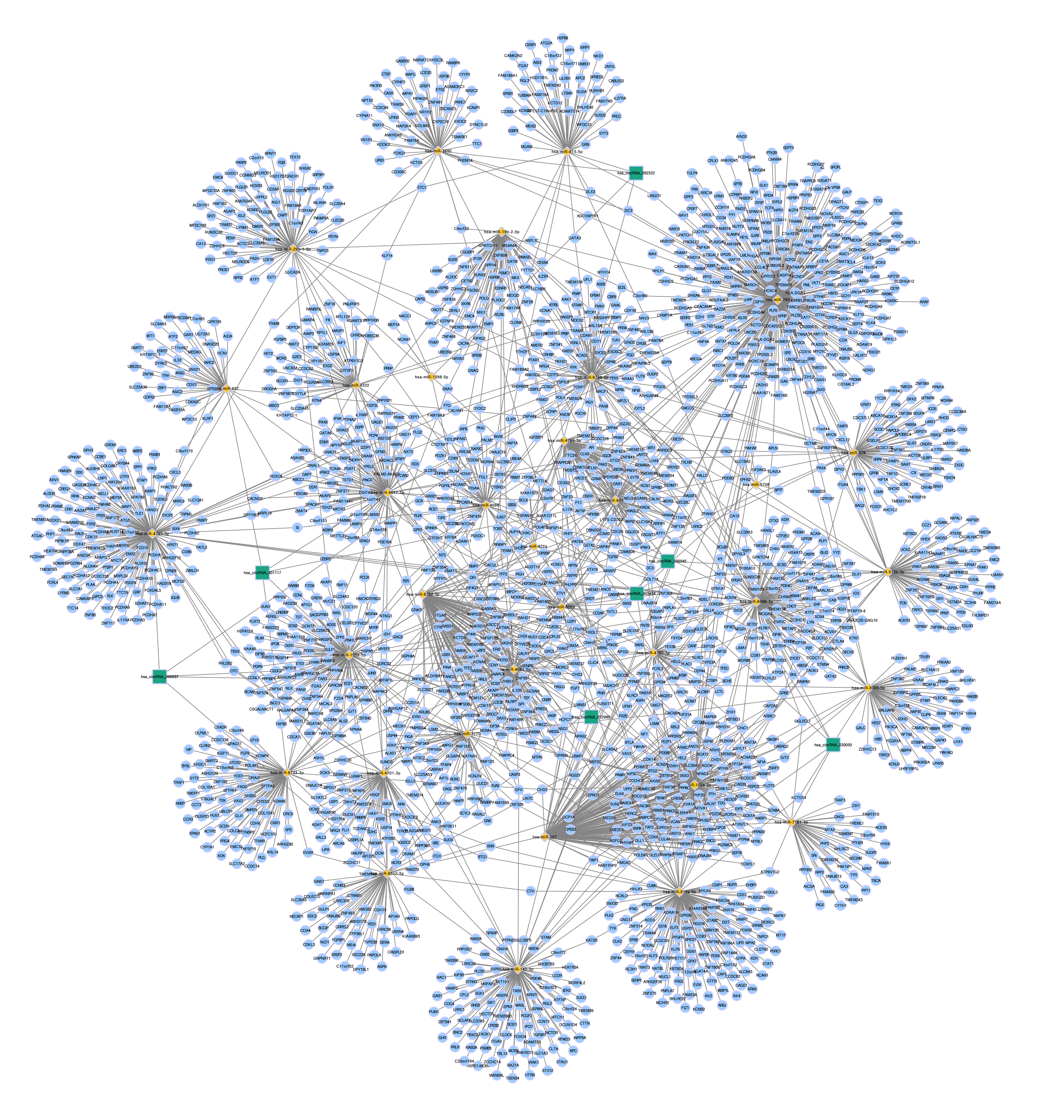
**

**Figure 6.** The representative circRNA-miRNA-mRNA network and sequence-pairing predictions for predicted circRNAs and miRNAs.
